# Interaction of a standardized mistletoe (*Viscum album*) preparation with antitumor effects of Trastuzumab in vitro

**DOI:** 10.1186/s12906-016-1246-2

**Published:** 2016-08-04

**Authors:** U. Weissenstein, M. Kunz, K. Urech, U. Regueiro, S. Baumgartner

**Affiliations:** 1 Iscador AG, Arlesheim, Switzerland; 2 Institute of Integrative Medicine, Witten/Herdecke University, Herdecke, Germany

**Keywords:** Mistletoe (*Viscum album* L.), Iscador, Trastuzumab, Herceptin, Her-2, Drug interactions, Cytostasis, Apoptosis, Cell cycle, VEGF

## Abstract

**Background:**

Besides conventional anticancer therapy many breast cancer patients use complementary and alternative medicine (CAM) like the medicinal herb mistletoe (*Viscum album* L.). To gain more knowledge about possible herb-drug interactions between CAM and conventional anticancer medications, in the present in vitro study we investigated the effect of a standardized mistletoe preparation on the action of Trastuzumab, a drug used for the treatment of Her-2 positive breast cancer.

**Methods:**

The Her-2 positive human breast carcinoma cell line SK-BR-3 was treated with Trastuzumab. Different doses of the drug were combined with *Viscum album* extract (VAE) in clinically relevant doses. Proliferation, apoptosis, cell cycle and the secretion of vascular endothelial growth factor (VEGF) were analyzed.

**Results:**

No inhibition of antitumor efficacy of Trastuzumab by VAE was detected. VAE and Trastuzumab, either alone or in combination, inhibited proliferation of SK-BR-3 cells in vitro. At higher concentrations VAE induced apoptosis, which was not observed for Trastuzumab. Cells treated with Trastuzumab underwent a G0/G1 cell cycle arrest and cells treated with VAE a G2/M arrest. After application of the two drugs in combination both G0/G1 and G2/M arrest was observed. VEGF secretion of SK-BR-3 cells was significantly inhibited by sole treatment with Trastuzumab or VAE. Combined treatment of Trastuzumab and VAE at clinically relevant doses showed additive inhibitory effects on VEGF secretion.

**Conclusions:**

VAE did not interfere with cytostatic effects of Trastuzumab on SK-BR-3 cells in vitro. Our in vitro results suggest that no risk of safety by herb drug interactions has to be expected from the exposition of cancer cells to Trastuzumab and VAE simultaneously. In contrast, VAE and Trastuzumab seem to exhibit complementary anti-cancer effects in vitro.

## Background

Breast cancer is the most common cancer in females worldwide with about 12 % of all new cancer cases and 25 % of all cancers in women [1, 2]. In 15–25 % of human breast cancers the HER2 receptor, encoded by the proto-oncogene ERBB2 is amplified. Her-2 overexpression has been correlated with poor clinical outcome. The selective, fully humanized recombinant monoclonal antibody (mAb) Trastuzumab (Herceptin) was developed to target HER2 with high affinity. It acts as an inhibitor of signal transduction and has been shown to decrease the proliferation of human tumor cells that overexpress HER2 both in vitro and in vivo [3, 4]. Besides primary and metastatic Her2 positive breast cancer, use of Trastuzumab is approved for the treatment of HER2-overexpressing metastatic gastric or gastroesophageal junction adenocarcinoma.

Breast cancer patients increasingly use CAM in addition to conventional therapy with the attempt to strengthen their immune system, prevent recurrence, to improve quality of life and feel more in control. With a prevalence of 40-80 % CAM use is high [5–7]. In Europe especially the application of mistletoe preparations is widespread [8–10]. Extracts derived from mistletoe (*Viscum album*) have been shown to display anti-tumoral, anti-angiogenetic and immune-potentiating activities [11–14]. Mistletoe preparations are used either alone or in combination with chemo-, radio- and hormonal and probably with new molecular targeted therapies to treat cancer or to lessen the side effects of anticancer drugs. Clinical studies suggest the association of mistletoe treatment with a better survival, a reduction of side effects of traditional therapies and with an increase of quality of life [15–18].

In a former in vitro study we demonstrated that mistletoe extracts did not influence the cytostatic and cytotoxic activity of several common conventional chemotherapeutic drugs when applied in concentrations typical for clinical use [19].

The aim of the present study was to investigate possible effects of clinically relevant doses of a standardized *Viscum album* extract (VAE) on the in vitro efficacy of Trastuzumab with regard to proliferation, apoptosis, cell cycle kinetics and production of vascular endothelial growth factor (VEGF) using the Her-2 positive cell line SK-BR-3.

## Methods

### Mistletoe extracts and drugs

The aqueous, fermented mistletoe preparation Iscador M spec. 5 mg (VAE, host tree *Malus domestica*, Lot 1109/2103/2, total mistletoe lectin concentration 287 ng/ml) was obtained from Iscador AG (Arlesheim, Switzerland). Vials of Iscador M spec. 5 mg, stored at 4 °C were used as VAE stock solution.

Trastuzumab (Herceptin®) (Lot. B4009) was kindly provided by Roche Pharma AG Switzerland. Stock solution (15 mg/ml in deionized water) was stored at - 20 °C.

### Cell culture

Human breast carcinoma cell line SK-BR-3 was obtained from DSMZ (German Collection of Microorganisms and Cell Cultures, Braunschweig, Germany).

SK-BR-3 cells were cultured in McCoy’s 5A Medium (Sigma-Aldrich) supplemented with 20 % FBS, 2 mM L-Glutamine and 1 % Penicillin - Streptomycin in a humidified atmosphere with 5 % CO_2_ at 37 °C. Cell line was maintained in exponential growth and cells from subconfluent monolayers were harvested by trypsin-EDTA (Sigma-Aldrich) to carry out the experiments. For measurement of the parameters, the cell cultures were used within 6–8 weeks after thawing.

### Proliferation assay

Proliferation was indirectly assessed using the cell proliferation reagent WST-1 (Roche, Mannheim, Germany). Cells (1.5 x 10^4^ in 100 μl) were plated in triplicates in 96-well plates. After 4–6 h to allow attachment, the drugs were added in various concentrations (see below). Proliferation rate was measured after 4 h of incubation with the WST-1 reagent in triplicate. The upper limit of absorbance was 2.0 - 2.1. Values are given in percent inhibition of proliferation relative to untreated control.

### Cell death analysis

Apoptosis/necrosis was measured using the Annexin V-FITC Apoptosis Detection Kit I (BD Biosciences Pharmingen™, San Diego, CA, USA). Briefly: 2 x 10^5^ cells were incubated with Annexin V-FITC and 7-AAD at room temperature in the dark. Thereafter, the samples were analysed in a flow cytometer (FACS Calibur, BD Biosciences, San Jose, CA). Early apoptotic cells: Annexin V-FITC positive and 7-AAD negative. Late apoptotic/necrotic cells: Annexin V-FITC positive and 7-AAD positive. Values are given in percent of total cell number.

### Cell cycle analysis

Cell cycle analysis was performed using the CycleTest™ Plus DNA reagent Kit (BD Biosciences, San Jose, CA) according to manufacturer’s instructions. DNA QC particles were used for quality control. Data were analyzed using the FlowJo 7.6.1 software (Ashland, OR, USA).

### Drug concentrations in the assays

Preceding the actual experiments the optimal concentration range and the optimal incubation time regarding treatment efficacy was determined for Trastuzumab using the WST-1 proliferation assay (data not shown). The concentration range for Trastuzumab was higher than the corresponding dose of 2-4 mg/kg used in clinic [20].

In the main experiments, Trastuzumab was added to culture medium at following concentrations: 0, 0.1, 1.0, 10 and 100 μg/ml. Depending on the assay, each dose of the drug was combined with 0, 0.1, 1.0, 10 or 100 μg/ml of VAE for measuring proliferation, apoptosis/necrosis, cell cycle and VEGF secretion. Typical clinical Iscador concentrations for subcutaneous application are 0.1 and 1 μg/ml, roughly corresponding to an injection of 5 mg Iscador when referring to the body weight and the amount of circulating blood, respectively. Concentrations of about 10 μg/ml correspond to doses used for intravenous Iscador applications. Parameters were measured after the appropriate incubation time.

### VEGF ELISA

2.5 x 10^5^ SK-BR-3 cells were plated in 6-well plates. After 4-6 h to allow attachment, the drugs were added in various concentrations in a final volume of 2 ml per well. Cell culture supernatants were collected after 3 days (3d) of incubation, centrifuged and stored in aliquots at −80 °C until analysis. VEGF-A concentrations were determined using a commercial Human VEGF ELISA kit (Sigma-Aldrich, MO, USA) according to manufacturer's instructions. The detection limit was 10 pg/ml.

### Data analysis

Three independent experiments were carried out for each combination of Trastuzumab and mistletoe extract. Data were analyzed with full 3-way analysis of variance (ANOVA, Type 6 decomposition) using Statistica 6.0 (Statsoft Inc., Tulsa, USA). For pairwise comparisons, the protected Fisher LSD-test was used. This procedure gives a good safeguard against false-positive as well as false-negative results [21]. Limit of significance was defined as *p* < 0.05.

## Results

### Proliferation

**Fig. 1:**
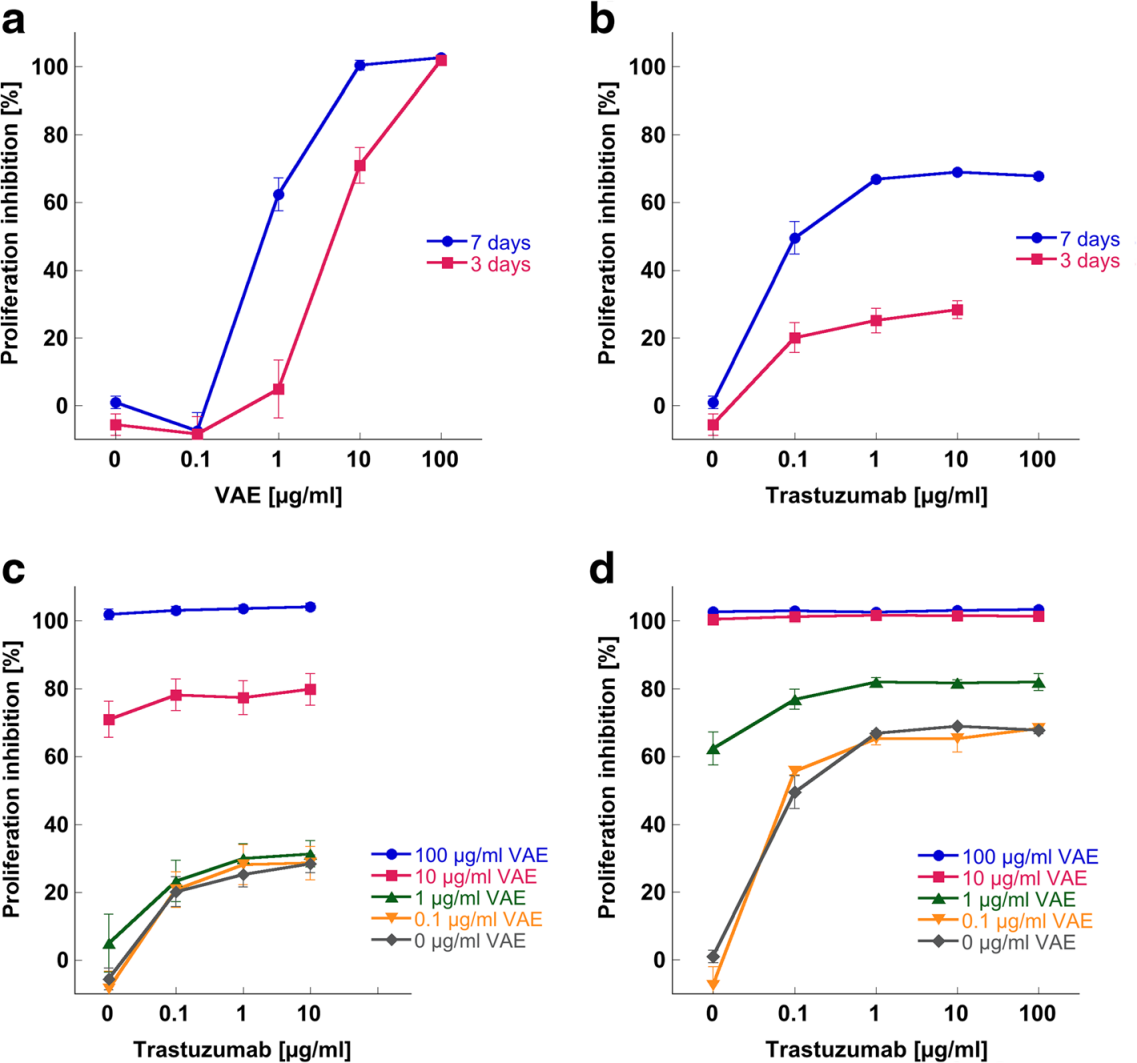
Dose response effect of VAE, Trastuzumab and their combinations on the proliferation of SK-BR-3 cells. Proliferation inhibition by (**a**) VAE treatment after 3d and 7d, (**b**) Trastuzumab treatment after 3d and 7d and combined VAE and Trastuzumab treatment after (**c**) 3d and (**d**) 7d, are shown. Cell growth kinetic was assessed with the WST-1 assay. Results are presented as mean ± SE from three independent experiments

The growth kinetic analysis of SK-BR-3 cells revealed different effects of VAE and Trastuzumab when used as single agents. A dose dependent anti-proliferative effect of VAE was observed at concentrations ≥10 μg/ml after 3d of incubation. After 7d a significant growth inhibition of 60 % with the clinically relevant concentration 1 μg/ml was detected and no proliferating cells were left at VAE concentrations of 10 and 100 μg/ml. 0.1 μg/ml VAE did not significantly affect the proliferation of tumor cells (Fig. 1a).

Figure 1b presents mean proliferation values of the breast carcinoma cell line SK-BR-3 treated with different concentrations of Trastuzumab. The maximal cytostatic effect attained by the treatment with Trastuzumab alone was 63 % after 7d of incubation. With 1 μg/ml Trastuzumab a plateau of inhibition was reached resulting in no further enhancement at concentrations of 10 and 100 μg/ml.

After simultaneous application of VAE and Trastuzumab, concentrations of 1–100 μg/ml VAE significantly enforced the antiproliferative effect of all Trastuzumab concentrations applied (*p* < 0.01), whereas 0.1 μg/ml did not show any modulation (Fig. 1c, d).

### Apoptosis

**Fig. 2:**
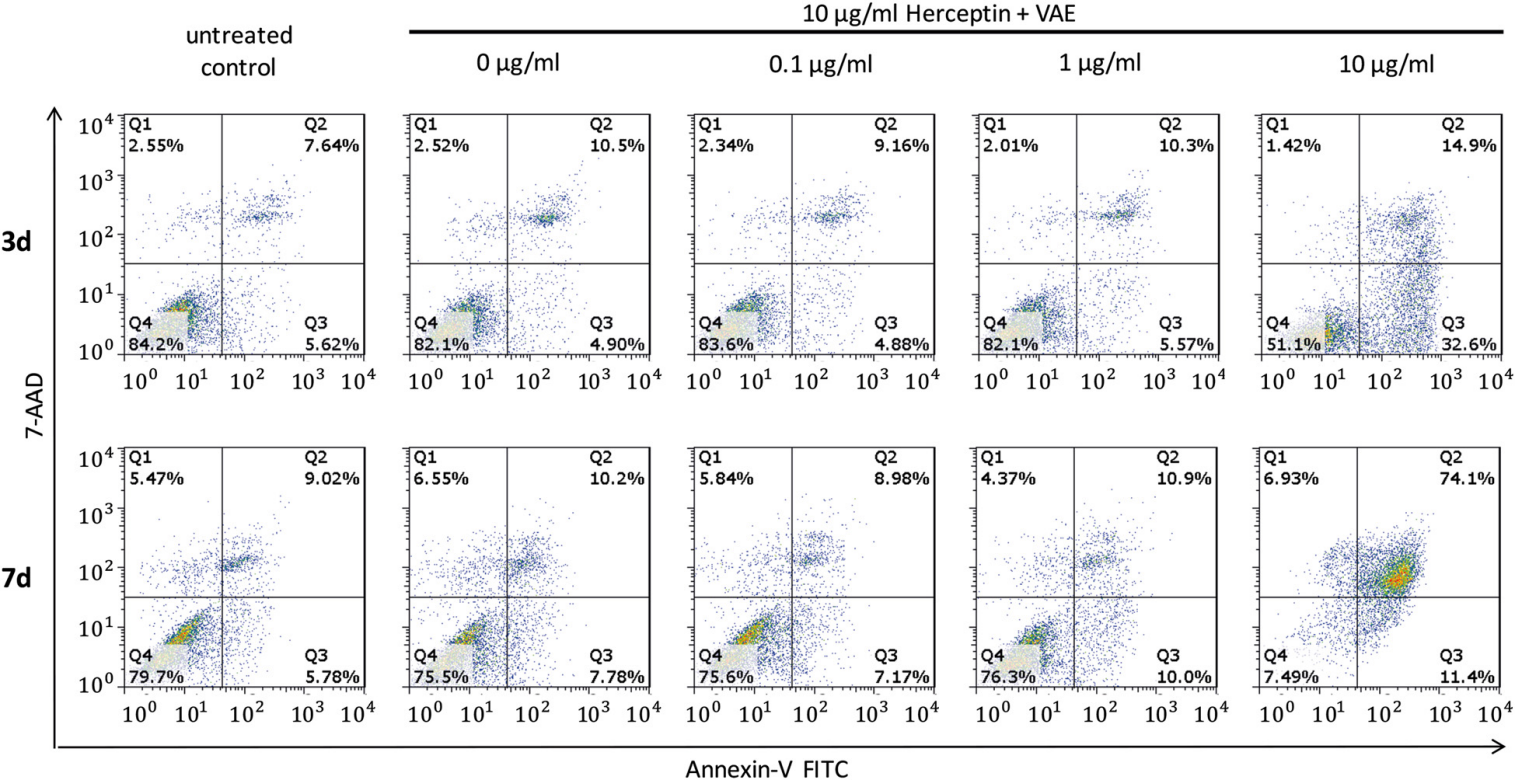
Apoptosis induction in SK-BR-3 cells by VAE. Flow cytometric analysis of apoptotic death in SK-BR-3 cells labelled with annexin-V FITC and 7-AAD after 3d (*top*) and after 7d (*bottom*) exposure to 10 μg/ml Trastuzumab in combination with 0 – 10 μg/ml VAE. This figure is representative of three independent experiments. The percentages in the graphs represent the percentage of cell numbers in each quadrant. Q1 and Q2: late apoptotic/necrotic cells, Q3: early apoptotic cells, Q4: living cells

To characterize the type of cell death induced by VAE and Trastuzumab, respectively, Annexin V/7-AAD dual staining was used to evaluate the apoptotic cells. VAE concentrations between 0.1 and 1 μg/ml had no significant effect on the viability of SK-BR-3 cells; VAE at 10 μg/ml induced apoptosis (*p* < 0.001, F-test, Fig. 2).

**Fig. 3:**
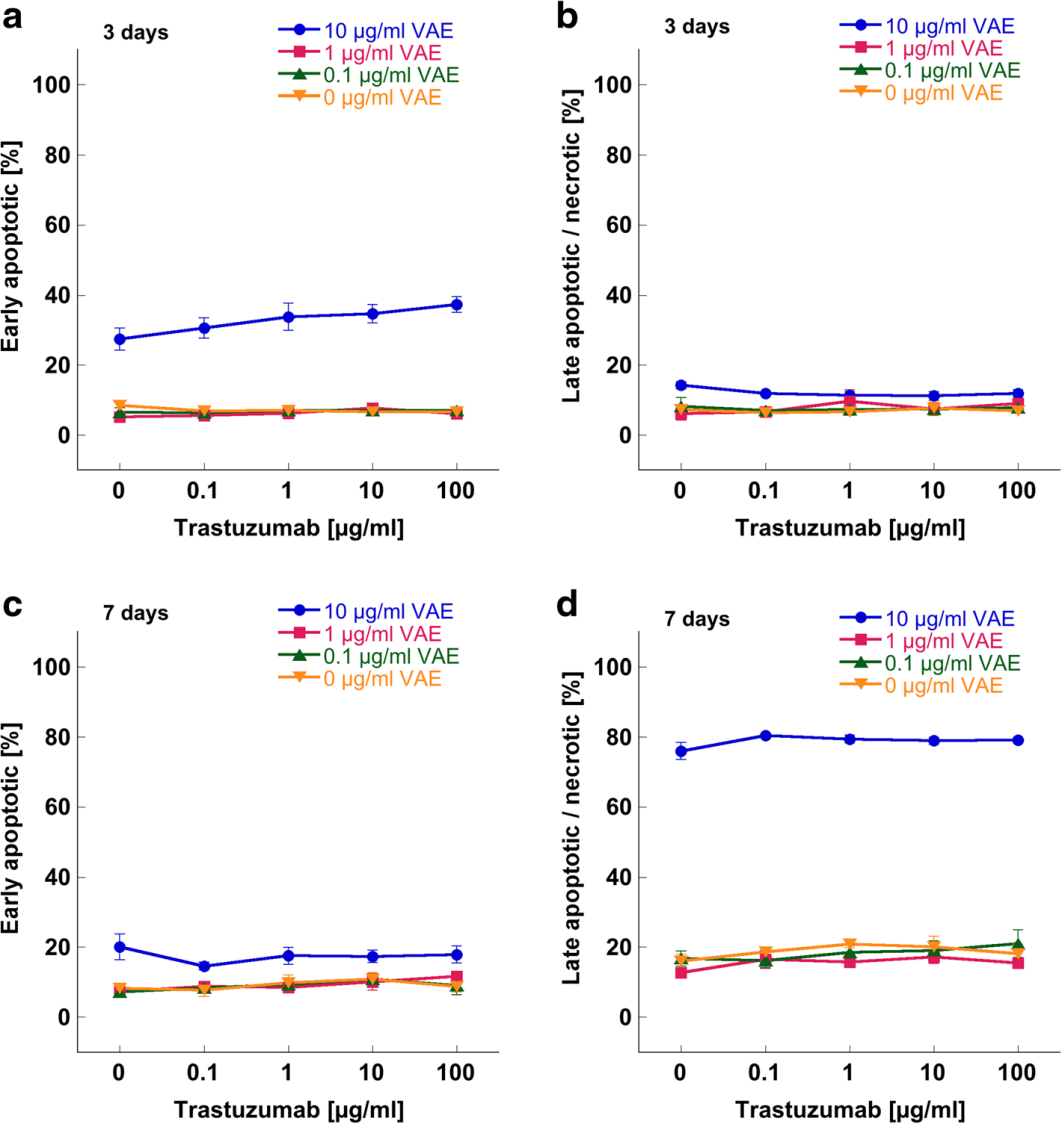
Apoptosis induction in SK-BR-3 cells. Mean values (±SE) of (**a**) early apoptosis and (**b**) late apoptosis/necrosis after 3d and of (**c**) early apoptosis and (**d**) late apoptosis/necrosis after 7d treatment with different concentrations of Trastuzumab combined with different concentrations of VAE are presented

With 10 μg/ml VAE after 3d the proportion of early apoptotic cells was elevated from 7.1 % in the untreated control to 32.8 % and that of late apoptotic/necrotic cells from 7.0 % in control to 12.1 % (Fig. 3a, b). After 7d the proportion of early apoptotic cells raised from 9.0 % in the control to 17.4 % and that of late apoptotic/necrotic cells from 18.7 % in control to 78.7 %, respectively (Fig. 3c, d).

In our experimental setup Trastuzumab alone did not exert a dose dependent pro-apoptotic or cytotoxic effect. After 3d and after 7d treatment of SK-BR-3 cells with concentrations of Trastuzumab between 0.1 and 100 μg/ml, the proportions of apoptotic or dead cells remained unaltered compared to the untreated control (*p* > 0.05, F-test) and Trastuzumab did not significantly alter VAE induced apoptosis (*p* > 0.8, F-test for interaction, Fig. 3), though there was a tendency that Trastuzumab enforced the pro-apoptotic effect of 10 μg/ml VAE after 3 days (Fig. 3a).

### Cell cycle analysis

**Fig. 4:**
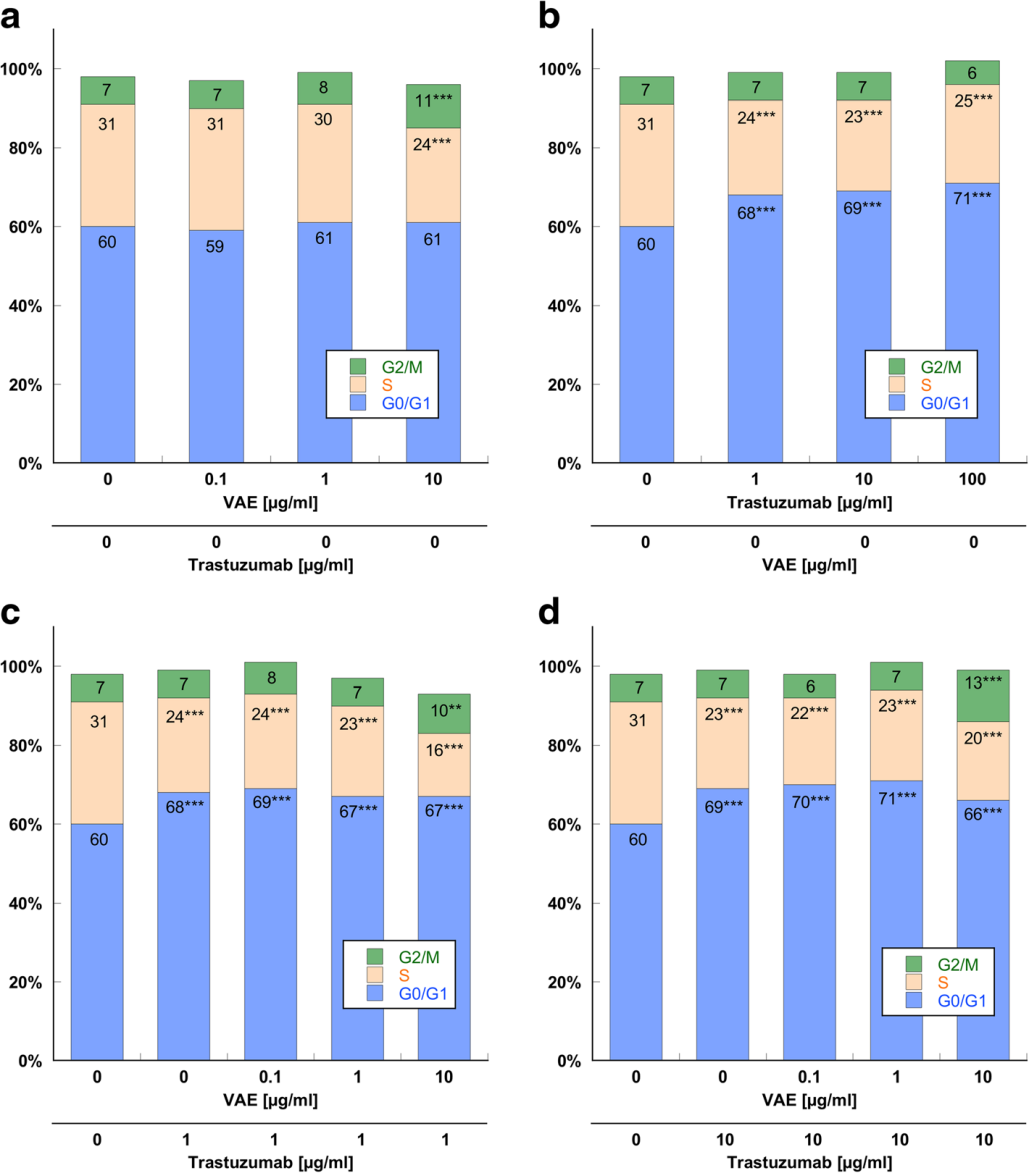
SK-BR-3 cell cycle analysis of cells treated with (**a**) VAE (**b**) Trastuzumab (**c**) 1 μg/ml Trastuzumab and VAE and (**d**) 10 μg/ml Trastuzumab and VAE. Cells were harvested after 3d, fixed, stained and analyzed for DNA content by flow cytometry. The distribution and percentage of cells in G0/G1, S and G2/M phase of the cell cycle are indicated. Results are presented as mean values from three independent experiments. (SE values are omitted for clarity). Significance values are given relative to the VAE and Trastuzumab untreated controls (**p < 0.01, ***p < 0.001). Discrepancies from 100 % can be attributed to slight gating differences and the display of mean values

VAE at 10 μg/ml influenced cell cycle kinetics by inducing a significant G2/M accumulation of 11 vs. 7 % in control (*P* < 0.001, Fig. 4a). The proportion of S-phase cells declined from 31 to 24 %. Lower VAE concentrations had no effect. VAE did not induce a G0/G1 accumulation at any concentration (*p* > 0.28, F-test).

As shown in Fig. 4b, Trastuzumab induced a dose dependent significant accumulation of SK-BR-3 cells in G0/G1 cell cycle phase (*p* < 0.001, F-test). The maximal percentage of cells found in G0/G1 phase was 71 % after treatment with 100 μg/ml Trastuzumab, compared to 60 % in the control. Trastuzumab did not induce a G2/M accumulation (*p* > 0.12, F-test).

Simultaneous treatment of cells with 10 μg/ml VAE and Trastuzumab induced a block in both cell cycle phases, G0/G1 (67 % with 1 μg/ml Trastuzumab and 66 % with 10 μg/ml Trastuzumab) and G2/M (10 % with 1 μg/ml Trastuzumab and 13 % with 10 μg/ml Trastuzumab) (Fig. 4c, d). VAE concentrations of 0.1 and 1 μg/ml did not influence the effect of Trastuzumab on cell cycle. There was no statistically significant interaction in the ANOVA model, which means that the effects of Trastuzumab and VAE were additive.

### VEGF production

**Fig. 5:**
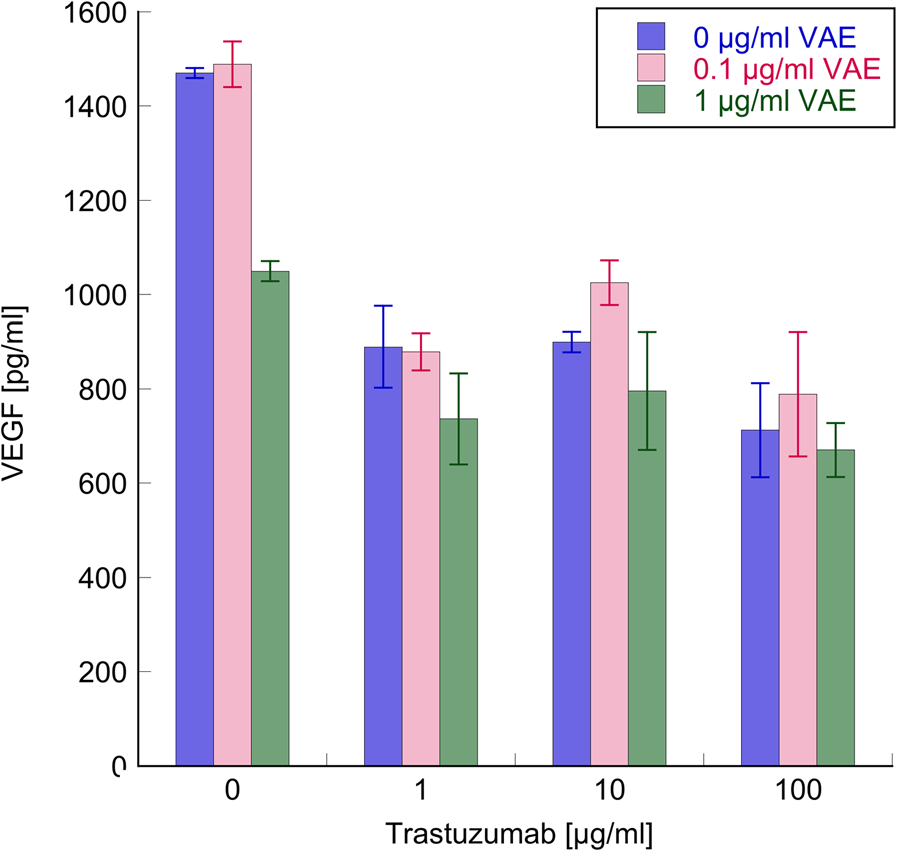
Inhibitory effect of Trastuzumab, VAE and their combinations on VEGF production of SK-BR-3 human breast carcinoma cells in vitro. VEGF was measured by ELISA in cell culture supernatants after 3d of treatment. Results are presented as mean ± SE from three independent experiments

We assessed whether treatment with VAE and/or Trastuzumab were able to reduce VEGF production in SK-BR-3 cells. Untreated SK-BR-3 cells were found to secrete appreciable quantities of VEGF (approximately 1500 pg/ml/2.5×10^5^ seeded cells) into the media. Both VAE and Trastuzumab reduced VEGF production (*p* < 0.001, F-test) without a significant interaction in the ANOVA model (*p* = 0.22), meaning that the effects on VEGF were independent (additive). As shown in Fig. 5, 1 μg/ml VAE reduced VEGF production to 71 % (*p* < 0.01), whilst Trastuzumab induced a decrease in VEGF production, which reached about 61 % at the concentrations of 1 and 10 μg/ml antibody and 48 % at the concentration of 100 μg/ml (*p* < 0.001 for all concentrations). Co-treatment with 1 μg/ml VAE and Trastuzumab revealed the most pronounced effects.

Proliferation inhibition is a possible underlying cause for lower VEGF concentrations in culture supernatants of treated cells. We investigated this hypothesis by calculating VEGF production relative to proliferation (Fig. 1) for the relevant concentrations of VAE and Trastuzumab. In this evaluation, application of 1 μg/ml VAE led to a significant reduction of VEGF production (86 %, *p* < 0.05); Trastuzumab did not yield any significant effects at any concentration applied (*p* > 0.15).

## Discussion

In our in vitro study we investigated the interaction between Trastuzumab, a monoclonal antibody used for the treatment of Her-2 positive breast cancer and an aqueous fermented mistletoe extract, frequently used by cancer patients as supportive therapy. No inhibition of antitumor efficacy of Trastuzumab by VAE was detected in any of our experimental setups. The same applies to the antitumor efficacy of VAE, which was not inhibited by Trastuzumab. When used individually, VAE and Trastuzumab showed dissimilar anti-proliferative effects. The growth inhibitory effect of Trastuzumab was associated with G0/G1 accumulation but not with apoptosis. VAE alone and in combination with Trastuzumab revealed a distinct pro-apoptotic activity and a G2/M arrest. Both Trastuzumab and VAE significantly inhibited VEGF production, alone as well as in combination.

Little is known so far about possible drug interactions of mistletoe preparations with Trastuzumab. In a retrospective case series study examining the tolerability of the simultaneous treatment with mistletoe preparations and Herceptin in nine breast cancer patients no new side effects that could be attributed to the well tolerated combined treatment were observed. However an increased incidence of mild reactions consisting of well-known side effects of either mistletoe or Herceptin treatment was noted and therefore a special attention was recommended (A.P. Simões-Wuest, personal communication). A prospective study would provide more conclusive information.

The aim of our study was to investigate if clinically relevant doses of VAE interfere with Trastuzumab in vitro by influencing its anti-tumoral efficacy on proliferation, cytotoxicity, cell cycle progression and VEGF production of a Her2 positive breast cancer cell line. Trastuzumab and VAE as well as their combinations induced dose and time dependent anti-proliferative effects on SK-BR-3 cells. Our results regarding growth inhibition and lack of apoptosis induction by Trastuzumab alone are comparable to data shown previously [22–25]. Depending on the treatment time VAE doses ≥1 μg/ml induced a 60 % and ≥10 μg/ml a total inhibition of cell proliferation. Concomitant treatment already with the clinically relevant dose of 1 μg/ml VAE could lead to an augmentation of Trastuzumab induced cytostasis for about 13-15 %.

Much of the Trastuzumab induced growth-inhibitory effects might be due to a transient cell cycle arrest. Consistent with former studies we observed a distinct G0/G1 accumulation in Trastuzumab treated SK-BR-3 cells [26, 27]. As an initial effect of specific antibodies on Her-2 receptor signaling, a redirection of cyclin-dependent kinase (Cdk) inhibitor p27 (also known as KIP1) onto Cdk2 complexes was proposed, resulting in the inhibition of G1/S progression. The p27-Cdk2 complex accumulation was correlated with reduced protein levels of D-type cyclins and transcription factor c-Myc [28].

Growth inhibition by higher VAE concentrations detected in our study could be strongly related to pro-apoptotic activity. Induction of apoptosis is a well-defined mechanism exerted by mistletoe lectins (ML). One of the constituents of VAE is ML-I, a heterodimeric ribosome-inactivating protein II (RIP II) composed of an A-chain with the capacity to depurinate a critical adenosine in the 28S ribosomal RNA and a sialic acid-specific B chain that binds to cell surfaces and facilitates internalization of the A-chain [29]. ML, like other type II RIPs induce cell apoptosis in vitro and in vivo probably via an intracellular pathways described as “ribotoxic stress response” that directly targets mitochondria and leads to apoptosis through a stress-mediated signalling pathway [30–32]. The ML-I induced apoptosis is reported to be receptor-independent. Cytochrome c is rapidly released from mitochondria into the cytosol accompanied by proteolytic activation of caspase-8, −9, −3 and −2 and decreased expression of anti-apoptotic molecules [33, 34]. If the ribosome inhibiting activity of ML or another mechanism or constituent is responsible for the proliferation inhibiting effect of 1 μg/ml VAE, a concentration were no apoptosis induction was observed, has to be evaluated.

VAE at 10 μg/ml induced a G2/M arrest and simultaneous treatment with Trastuzumab and 10 μg/ml VAE induced the accumulation of SK-BR-3 cells in both G0/G1 and G2/M cell cycle phases. The G2/M phase arrest with a decline in the proportion of S-phase cells after treatment with VAE was observed in parallel to the rise of apoptotic cell content. It is known, that the processes of cell cycle progression and programmed cell death use and control shared factors like p53, p21, RB, c-Myc and several cyclin-dependent kinases (Cdks) and their regulators [35]. Activated p53 can cause apoptosis, G0/G1 arrest but also functions at the G2/M checkpoint [36] but in breast cancer SK-BR-3 cell line the p53 gene is mutated [37]. The investigation of recombinant ML effects on tumor cell proliferation in p53-wild-type and -deficient murine embryo fibroblasts indicated a predominantly p53-independent mechanism of apoptosis induction by rML [38]. Changes in cell cycle may be as well a p53-independent secondary effects of ribosome inhibition and ribotoxic stress by VAE that block entry into mitosis. G2/M arrest and apoptosis can result from upregulation of p53, p21, Chk1 and Chk2 (checkpoint kinases 1 and 2) and downregulation of cyclin B1 or cyclin D1 [35, 39, 40]. Miyoshi et al. demonstrated a marked elevation of p21 mRNA and protein upon exposure of a human myeloid cell line to ML (VAA, *Viscum album* agglutinin) [41]. P21 participates in the maintenance of cells in G1-phase arrest but also in G2/M through multiple mechanisms [42, 43]. In squamous cell carcinoma cell lines VAE treatment led to a substantial decrease in the expression level of Cyclin D1 [44]. Cyclin D1 performs a critical cell cycle regulatory function during G2 phase [45] and silencing of Cyclin D1 also had an effect on apoptosis induction in several squamous cell carcinoma cell lines [46].

In summary our results suggest that Trastuzumab and VAE influenced different cell cycle regulators in SK-BR-3 cells whereby the G2/M arrest induced by VAE seemed to be correlated to apoptosis related events. Molecular investigations could provide further confirmation on this subject.

One of the fundamental physiological processes of tumor growth is angiogenesis, mediated by pro- and anti-angiongenic factors [47, 48]. Her-2 signaling affects the expression of angiogenic factors like VEGF, Interleukin-8 and thrombospondin-1 [49]. VEGF stimulates endothelial cell proliferation and migration and causes the degradation of the basement membrane in microvessel walls. In cancer cells VEGF is supposed to promote proliferation, survival and invasiveness [50, 51]. In our experiments we demonstrated a significant inhibition of VEGF production by SK-BR-3 cells treated with Trastuzumab. Concomitant treatment with VAE at clinically relevant doses did not negatively affect the inhibitory efficacy of Trastuzumab, co-treatment with 1 μg/ml VAE revealed even lower VEGF values. In SK-BR-3 cell cultures treated with Trastuzumab the inhibition of VEGF correlated with growth reduction and seemed not to be a primary effect. Several in vivo and in vitro studies demonstrated that the treatment with Her-2 neutralizing antibodies like Trastuzumab induced a down-regulation of VEGF mRNA and protein levels in Her-2-overexpressing tumors resected from xenografted mice as well as in Her-2 overexpressing breast tumor cell lines, associated with inhibition of tumor growth and vessel formation [49, 52].

Sole VAE caused a significant VEGF diminution that was mainly independent of proliferation inhibition what indicates a direct suppressive effect of VAE on VEGF expression besides the reduction of VEGF protein content in cell culture media due to lower cell densities. If this effect is specific or a consequence of ribosome inhibiting events has to be proven. Varying results on the effect of mistletoe on angiogenesis exist. Inhibitory impact of VAE on vessel development in vitro and in vivo was associated with apoptosis induction [53] but in another study VAE-treated glioblastoma cells down-regulated central genes involved in glioblastoma progression and malignancy, among others VEGFA, without parallel cytotoxicity and apoptosis [54].

In our in vitro study we intended to contribute information about possible interactions of VAE with Trastuzumab. We addressed some of the mechanisms of action proposed for Trastuzumab [55] using a Her2 positive breast cancer cell line. One of the limitations of working with cell lines is, that interaction and protection mechanisms otherwise available from the donor organism are eliminated. Although we could not detect pro-apoptotic activity of Trastuzumab in our experiments it has been shown that neoadjuvant treatment with Trastuzumab in vivo induced some apoptosis in primary breast cancers [56]. In addition to the direct effects on cancer cells, there was evidence that antibody-dependent cellular cytotoxicity (ADCC) plays an important role in the antitumor activity of Trastuzumab, a mechanism we could not explore in our in vitro experiments. In vivo, specific immune cells could recognize and lyse tumor cells coated with the antibody. As mistletoe was reported to exert immune-modulating activities that may enhance the host defense system against tumors it would be interesting to investigate if ADCC could be fostered by mistletoe co-treatment.

## Conclusions

Aqueous, fermented mistletoe extract did not attenuate the anti-tumor activity of Trastuzumab in vitro when applied in concentrations typical for clinical use. We investigated the effects of VAE concomitant to Trastuzumab on proliferation, apoptosis induction, cell cycle progression and VEGF expression in a Her-2 overexpressing breast cancer cell line and could show concentration dependent additive effects. Our in vitro results suggest that VAE can be used without impairment of Trastuzumab efficacy. Further studies should be performed to complete the knowledge about drug interactions and patient safety and to investigate if the positive additive effects by VAE treatment concomitant to Trastuzumab could also be found in vivo.

## Abbreviations

ADCC antibody-dependent cellular cytotoxicity; CAM complementary and alternative medicine; Cdk cyclin-dependent kinase; EDTA Ethylenediaminetetraacetic acid; FBS Fetal bovine serum; ML mistletoe lectin; RIP II ribosome-inactivating protein II; rML recombinant mistletoe lectin; VAE *Viscum album* extract; VEGF vascular endothelial growth factor; WST-1 water soluble tetrazolium-1
